# Viral Evolution in the Genomic Age

**DOI:** 10.1371/journal.pbio.0050278

**Published:** 2007-10-02

**Authors:** Edward C Holmes

**Affiliations:** Princeton University, United States of America

## Abstract

The remarkable increase in the number of viral genome sequences represents both opportunities and challenges for understanding disease ecology and evolution, and must stimulate researchers to address questions that were previously considered out of reach.

Genome sequence data will undoubtedly deliver much to the study of viral pathogens and their diseases. A prominent example of this new genomic perspective is influenza A virus, for which a large-scale genome sequencing project begun in the year 2005 has, to date, generated around 2,500 complete viral genomes [1]. While this alone is newsworthy, the rise of rapid, high-throughput genome “pyrosequencing” promises to take the production of viral genomes to a level once unimaginable [2].

Yet advances in genome sequencing also create a major intellectual challenge; rather than simply maintaining ever-larger genomic databases for relatively straightforward surveys of viral biodiversity and molecular epidemiology, it is crucial that we direct the power of genomics to help address questions of more fundamental biological importance. Genome sequence data has the potential to shed new light on many key questions in viral evolution and epidemiology, and here I outline three research avenues where the large-scale comparison of genome sequences will be of particular importance.

## Interactions among Pathogens

Evolutionary studies of viral pathogens have, with few exceptions, tended to focus on individual species. If an attempt is made to place their evolution “in context,” this usually only relates to the different host species that a virus infects. For example, there is currently great interest in determining the viral and host determinants for the sustained transmission of H5N1 influenza A virus in birds as opposed to humans. Although such studies are an essential part of modern molecular epidemiology, an exploration of how the multiplicity of pathogens that co-circulate within a single host population might influence each other's evolution and etiology is strikingly absent. Similarly important questions include: What role does cross-protective immunity play in shaping microbial diversity? How widespread is ecological interference among pathogens?

Existing data already hint at the importance of evolutionary interactions among pathogens. For example, the HIV pandemic has resulted in an abundance of people with pronounced immune deficiency, stimulating a resurgence in opportunistic pathogens like Mycobacterium tuberculosis. It is also possible that widespread immunodeficiency will assist the emergence of new pathogens [3], perhaps by extending the infectious period of normally acute viral infections. Similarly, the nature of the interactions among the four serotypes of dengue virus has been a subject of much debate, particularly whether immunological responses to different serotypes are usually cross-protective [4] or enhancing [5]. Not only might these interactions dictate underlying patterns of genome evolution [6], but they will evidently have a major bearing on successful vaccination.

Finally, it has long been known that influenza-associated mortality is largely due to secondary pneumonia caused by Streptococcus pneumoniae bacteria [7]. However, although we now have a wealth of data on the genetic diversity of influenza virus in both time and space, there has been no attempt to tie these evolutionary patterns with those of the co-infecting bacterial population. Influenza virus is also just one of the respiratory pathogens that circulate in human populations, with other notables including parainfluenza virus, respiratory syncytial virus, and the abundant rhinoviruses. Despite the disease burden due to these viruses, little is known about how they interact at the evolutionary and epidemiological scales. The comparative analysis of their genome sequences, in which changes in genetic diversity (or phylogenetic structure) in one virus are placed in the context of the contemporaneous evolutionary patterns and processes exhibited by co-circulating pathogens, may provide a valuable way to study their interactions.

## Linking Evolutionary Change at the Intrahost and Interhost Scales

Large population sizes, rapid replication, and extremely high mutation rates mean that populations of RNA viruses usually harbor extensive genetic diversity [8]. Despite this, the vast majority of studies of genetic diversity in RNA viruses, particularly for acute infections, have been conducted at the epidemiological level, in which a single consensus sequence is generated from each infected individual. This sequence must then describe the average diversity in the intrahost viral population, masking myriad variable mutant sequences, some of which may have a major bearing on fitness. However, determining the extent and structure of intrahost genetic variability and how it relates to that observed at the epidemiological scale is of fundamental importance for understanding many aspects of evolutionary dynamics, including the likelihood of successful cross-species transmission and emergence [9].

For studies of RNA virus evolution to truly come of age, it is critical that the relationship between intra- and interhost evolution be explored in depth. Major questions include: What is the fitness distribution of mutations sampled from within hosts? Do the processes of intra- and interhost differ in fundamental ways? What proportion of intrahost diversity is passed between hosts at transmission? Thankfully, the barriers of time and cost that prohibited studies of this kind in the past have now largely been dismantled in the age of genomics. Experimental infections may represent a particularly profitable research avenue in which intrahost evolution is documented in samples collected every few days (or even hours), and also allowing viruses to be passed among hosts, thereby providing a window on the dynamics of interhost transmission.

## Genome-Wide Interactions

If there is a lesson to be learned from the history of population genetics, it is that the more fine-scaled the data available for analysis—from allozymes to genomes—the more powerful the biological inference. Not only does the comparison of complete genomes invariably provide greater resolution of the spatial and temporal dynamics of viral spread, but it obviously enables the study of genome-wide interactions. As a case in point, the complex evolutionary processes that underpin the recent dramatic rise of resistance to adamantane drugs in influenza A virus, including the central role played by epistasis, were not revealed until an analysis of complete genome sequences was undertaken [10]. Rather than focusing on single genes in isolation, it is therefore essential that we examine the similarities and differences in evolutionary patterns among all the genes in a viral genome.

Although many microbes would benefit from large-scale genomic comparisons, it is striking that the most common human viruses, including the respiratory viruses mentioned above and the diarrhea-causing rotaviruses, are distinguished by the least amount of available genome sequence data. It is hoped that the influenza virus genome project, where the added value of genomic data has been amply demonstrated, will serve as model for other viral pathogens. Some key questions within this research agenda include: How frequent, and of what type, are the epistatic interactions among genes? What role does epistasis play in the development of drug resistance and immune escape? Do viral antigens drive the evolution of viral genomes as a whole?

## The Challenge of Genome Data

Despite the wealth of evolutionary and epidemiological data contained within viral genomes, there is little doubt that the availability of computational tools to analyze such an enormous data resource represents a major obstacle to those working at the interface of genomics and bioinformatics. Indeed, we are now entering the age where effective data analysis, rather than data availability, is set to become the major factor limiting progress. To date, most comparative studies of viral populations have considered tens, and at most a few hundred, of gene sequences. However, the rise of pyrosequencing means that the in-depth analysis of many thousands of genomes is now essential. Further, and perhaps more importantly, the power of genomic data is only truly realized if they are combined with detailed functional, experimental, and epidemiological information. As such, the analysis of viral genome data not only requires tools for sequence manipulation but those that can associate these data with a wider range of biological variables.

The remarkable increase in the number of viral genome sequences represents both opportunities and challenges to those working in the arena of disease ecology and evolution. Rather than being overwhelmed by the scale of the data that will characterize the genomic age, we must let it stimulate us to address questions that were previously considered out of reach.
